# Supplementary material for: Detection of Colorectal Cancer and Advanced Adenoma by Liquid Biopsy (Decalib Study): The ddPCR Challenge
Source: Cancers (Basel). 2020 Jun 6;12(6):1482. doi: 10.3390/cancers12061482 (PMC7352444; doi:10.3390/cancers12061482)
Supplement: Supplementary file 1 [file cancers-12-01482-s001.zip › cancers-814900-supplementary/Supplementary data modified/cancers-814900 supplementary figures_GT.pdf]

cancers

Supplementary materials: Detection of Colorectal Cancer and Advanced Adenoma by Liquid Biopsy (Decalib Study): The ddPCR Challenge

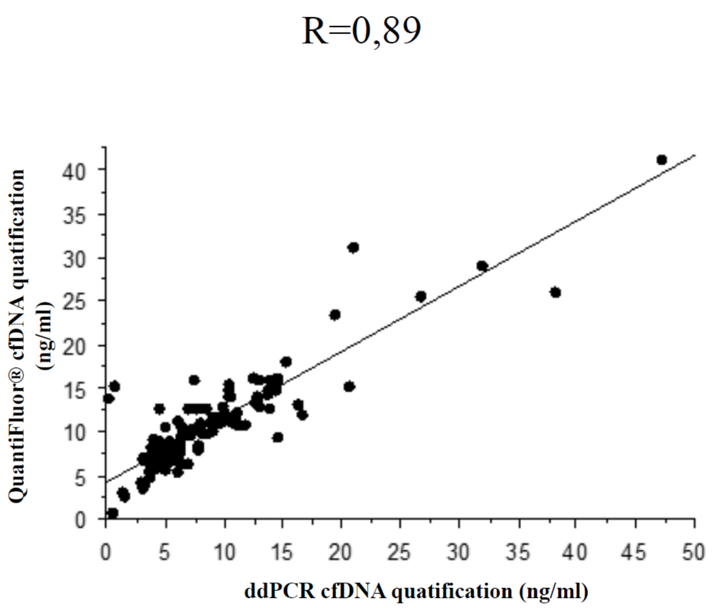

Figure S1. Comparison between the two methods of cfDNA quantification (ddPCR and QuantiFluor®).

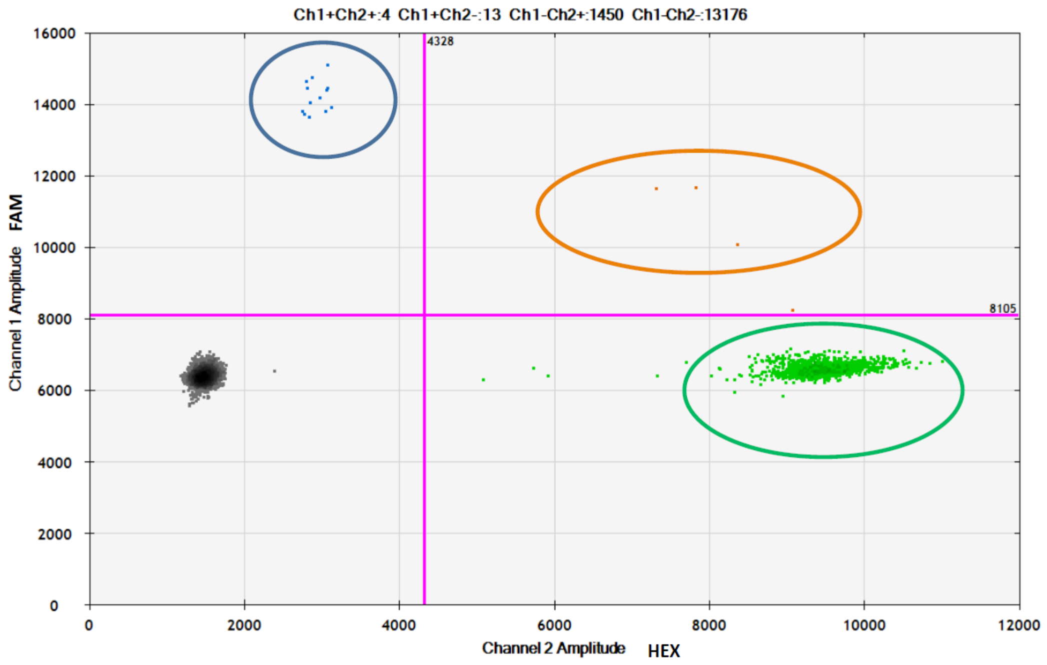

Figure S2. Representative 2D plots of the droplet digital PCR profile of a *BRAF V600E*-mutated control cell line. The pink lines indicate the threshold. Blue droplets are FAM positive droplets therefore containing mutated target DNA. Green droplets are HEX positive droplets containing the wild-type (non-mutated) target DNA. The FAM+HEX droplets are orange and contain the two types

of target DNA (mutant + wild-type). Finally, empty droplets do not present any fluorescent signal therefore are represented in grey.
